# GinExtraMed: Focus on *Rosa canina* L. Extract Encapsulated into Glycethosomes and Allanthosomes for Accelerating Skin Wound Healing

**DOI:** 10.3390/pharmaceutics17050632

**Published:** 2025-05-09

**Authors:** Martina Rossi, Simone Rossello, Valentina Sallustio, Manuela Mandrone, Teresa Cerchiara, Ilaria Chiocchio, Giuseppe Chidichimo, Michele Protti, Laura Mercolini, Barbara Luppi, Federica Bigucci, Angela Abruzzo, Concettina Cappadone

**Affiliations:** 1 Drug Delivery Research Laboratory, Department of Pharmacy and Biotechnology, Alma Mater Studiorum, University of Bologna, Via San Donato 19/2, 40127 Bologna, Italy; martina.rossi12@unibo.it (M.R.); simone.rossello2@unibo.it (S.R.); valentina.sallustio2@unibo.it (V.S.); barbara.luppi@unibo.it (B.L.); federica.bigucci@unibo.it (F.B.); angela.abruzzo2@unibo.it (A.A.); 2 Pharmaceutical Biochemistry Laboratory, Department of Pharmacy and Biotechnology, Alma Mater Studiorum, University of Bologna, Via San Donato 19/2, 40127 Bologna, Italy; concettina.cappadone@unibo.it; 3 Center for Applied Biomedical Research (CRBA), Alma Mater Studiorum, University of Bologna, Via Massarenti 9, 40126 Bologna, Italy; 4 Pharmaceutical Botany Laboratory, Department of Pharmacy and Biotechnology, Alma Mater Studiorum, University of Bologna, Via Irnerio 42, 40126 Bologna, Italy; manuela.mandrone2@unibo.it (M.M.); ilaria.chiocchio2@unibo.it (I.C.); 5 Department of Chemistry and Chemical Technologies, Università della Calabria, Via P. Bucci, 87036 Arcavacata di Rende, Italy; giuseppe.chidichimo@unical.it; 6 Pharmaco-Toxicological Analysis (PTA Laboratory), Department of Pharmacy and Biotechnology, Alma Mater Studiorum, University of Bologna, Via Belmeloro 6, 40126 Bologna, Italy; michele.protti2@unibo.it (M.P.); laura.mercolini@unibo.it (L.M.)

**Keywords:** *Rosa canina* L. extract, Spanish broom, wound dressings, encapsulation, glycethosomes, allanthosomes, skin wounds, scratch test

## Abstract

**Background/Objectives:** Over the last decade, the development of innovative wound products has continued to be a focus of intense research to meet the huge demand of patients. The aim of this work was to develop novel medicated Spanish broom wound dressings capable of releasing *Rosa canina* extract, recognized for its high antioxidant activity. **Methods:** *Rosa canina* extract was encapsulated in two different nanocarriers, namely glycethosomes and allanthosomes. The physico-chemical and functional characteristics of the obtained vesicles were described, including their size, particle size distribution, ζ potential, and encapsulation efficiency (EE). In addition, vesicles cytotoxicity and cell proliferation were evaluated on human fibroblasts. Furthermore, loaded vesicles were sunk into Spanish broom dressings, analyzed by confocal microscopy, and, finally, evaluated for their wound healing ability by scratch test. **Results:** Both carriers are nanometric in size, with a good EE (>70%), and a negative ζ potential. Additionally, vesicles are biosafe, non-cytotoxic, and lead to complete closure of the scratch in about 30 h. **Conclusions:** The findings showed that the developed Spanish broom dressings have the potential to be an efficient and innovative wound care product for accelerating skin wounds.

## 1. Introduction

Generally, the health condition of people depends on various factors, including the well-being of the skin. However, events such as wounds, trauma, inflammatory disorders caused by distinct agents, and microbial infections can lead to serious consequences, as well as significant health and economic impact [1,2]. Concerning wounds, there is a significant effort and commitment by researchers to develop innovative wound dressings able to restore skin integrity, to reduce healing time, and to decrease the economic and social costs for healthcare institutions and professionals, patients, and their families [3]. Nowadays, wounds’ physiology and the healing process have been well described in the literature [4,5,6]. Specifically, wounds are breaks in the skin are often caused by a blow or cut, and the healing process involves four general phases: hemostasis, an inflammatory response, proliferation, and remodeling [7]. The appropriate management of wounds, including the choice of the proper wound dressings, must occur from the beginning of healing process. Therefore, it is important to select multifunctional green biomaterials that can restore skin integrity [8]. The aim of our research project GinExtraMed, which has been supported by *Alma Mater Studiorum* University of Bologna, was to develop a multifunctional (antioxidant and healing) wound dressing based on green biomaterials such as Spanish broom fibers and *Rosa canina* extract (RC), according to the concept of sustainability.

Spanish broom (*Spartium junceum* L.), known in Italy as Ginestra, is a typical and spontaneous species of the Mediterranean area, where it forms dense patches growing on poor and infertile soils. It colonizes abandoned agricultural lands and rangelands [9], and is also able to modify the chemical composition of the soil, increasing its fertility [10]. Spanish broom has attracted significant attention thanks to its good physical and chemical characteristics that provide several advantages, including ready availability from renewable natural sources and biodegradability. Moreover, Spanish broom could be included in the list of plant fibers (cotton, jute, flax, and hemp) that are used frequently in wound management, as reported in our previous works [11,12].

To accelerate the wound healing process, Spanish broom fabrics can be impregnated with plant extracts, whose well-known efficacy in skin injuries can be attributed to the presence of bioactive chemicals such as flavonoids, phenols, alkaloids, and triterpenoids [13]. The final products exhibit antibacterial and antioxidant properties, improve collagen deposition, and promote proliferation of fibroblasts and keratin-forming cells [14,15].

Among plant extracts, we selected *RC* extract, used in traditional European folk medicine thanks to its anti-inflammatory and antioxidant activity [16]. *Rosa canina* L. belongs to the Rosaceae family, and is a shrub widespread in Europe. Polyphenols and ascorbic acid are the main bioactive compounds showing potential health benefits. However, these antioxidant compounds are not stable to temperature, light, and oxygen action, and their antioxidant activity could be easily lost [17,18]. To overcome these limitations, a valid and efficient approach is represented by the encapsulation in nanocarriers (liposomes, phytosomes, ethosomes, niosomes, and transferosomes) [19]. To our knowledge, in this work, for the first time, *RC* extract was encapsulated in two different vesicles, namely glycethosomes (GlyEts) and allanthosomes (AllEts). Specifically, GlyEts are phospholipid vesicles hydrated by a mixture of water, glycerol, and ethanol, while AllEts are phospholipid vesicles hydrated by a mixture of water, allantoin, and ethanol. Both vesicles were characterized for their physico-chemical and functional properties, such as size, particle size distribution, ζ potential, and encapsulation efficiency. In addition, vesicle cytotoxicity and cell proliferation induction were evaluated through 3-(4,5-dimethylthiazol-2-yl)-2,5-diphenyl tetrazolium bromide (MTT) assay and cell cycle analysis on 3T3 fibroblasts, respectively. Finally, loaded vesicles were sunk into Spanish broom dressings and a wound healing scratch test was evaluated.

## 2. Materials and Methods

### 2.1. Materials

Rosehips were collected in November 2022 from wild plants in Montefalcone nel Sannio (CB, Molise, Italy), GPS coordinates 41°51′29.6″ N 14°36′55.2″ E. A voucher specimen was deposited in the herbarium of Bologna University Botanical Garden with the code BOLO0602029. Lipoid H50 (fat free sunflower Lecithin with 60% phosphatidylcholine, non-GMO) was gifted from Lipoid HmbH (Ludwigshafen, Germany). Ethanol and methanol (purity > 99.8%) and all the solvents were purchased from Sigma-Aldrich (Milan, Italy), as well as 2,2-diphenyl-1-picrylhydrazyl radical (DPPH) and Calcium carbonate. Folin–Ciocalteu reagent was purchased from Titolchimica (Pontecchio Polesine, Italy). Vegetable glycerol 85% and allantoin were purchased from FarmaLabor (Canosa di Puglia, Italy) and Polichimica S.R.L (Bologna, Italy), respectively. Spanish broom dressings were provided by Prof. Giuseppe Chidichimo of the University of Calabria (Arcavacata di Rende, CS, Italy). Dulbecco’s modified Eagle medium supplemented with 4.5 g/L of D-glucose was purchased from Sigma-Aldrich Co. (St. Louis, MO, USA), as well as 3-(4,5-Dimethylthiazol-2-yl)-2,5-Diphenyltetrazolium Bromide (MTT) reagent. Fetal bovine serum (FBS), L-glutamine, penicillin, and streptomycin were purchased from Euroclone S.p.a. (Milan, Italy). WS1 human dermal fibroblasts were purchased from ATCC (American Type Culture Collection ATCC, Manassas, VA, USA). Tert-butylhydroperoxide (TBH) was purchased from Acros-organics (Geel, Belgium), and 2HDCF-DA was purchased from Sigma-Aldrich (Milan, Italy). Finally, Vybrant™ DiO Cell-Labeling Solution was purchased from Thermo Fisher Scientific Inc. (Waltham, MA, USA).

### 2.2. Preparation of Rosa canina Extract

To prepare a hydroalcoholic extract, 20 g of the fresh rosehip pulp was added to 150 mL of a 50:50 (*v*/*v*) ethanol/water mixture and subjected to sonication using a Transonic TP690 (Elma, Singen, Germany) for 40 min at room temperature [20]. The mixture was centrifuged at 4000 rpm for 20 min, and the supernatants were filtered using a Buchner filter (Rotofix 32A by Hettich, Tuttlingen, Germany). The residue was extracted again by repeating the procedure mentioned above. The collected supernatant was evaporated under vacuum at 40 °C using Buchi Rotavapor Heating Bath B-490 to remove alcohol and most of the water. The concentrated extract was subsequently freeze-dried in a Christ Freeze Dryer ALPHA 1-2 (Milan, Italy) at 0.01 atm and at −48 °C for 72 h. The lyophilized extract was stored at 4.0 ±1.0 °C in the dark until use.

### 2.3. Chemical Analysis of Rosa canina Extract

#### 2.3.1. HPLC-DAD-MS/MS Analysis of Bioactive Compounds in *Rosa canina* Extracts

Rosehip extracts were analyzed exploiting an HPLC-DAD-MS/MS method as outlined in our previous study [21]. Analyses were performed on a Waters (Milford, MA, USA) Alliance e2695 LC system, coupled with a Waters 2998 diode array detector and a Waters Micromass Quattro Micro triple quadrupole mass spectrometer. Separation was achieved on a Restek (Bellefonte, PA, USA) Ultra AQ RP C18 column (50 mm × 2.1 mm; 3 μm). Mass spectrometry data were acquired in multiple reaction monitoring (MRM) mode by means of an electrospray ionization source in negative polarity (ESI-). Due to the physicochemical properties of the analytes, two different analytical conditions were employed: one for phenolic compounds (cyanidin, gallic acid, (+)-catechin, and quercetin) and another one for ascorbic acid quantitation. The injection volume for both systems was 10 μL. For phenolic compounds, the flow rate was 400 μL/min, and the mobile phase components were 0.1% (*v*/*v*) formic acid (FA) in acetonitrile (component A) and 0.1% (*v*/*v*) FA in ultrapure water (component B), while the mobile phase composition gradient was: 0 min, 2% A; 3 min, 2% A (isocratic); 5 min, 15% A (linear gradient); 9 min, 15% A (isocratic); 14 min, 2% A (linear gradient); 16 min, 2% A (isocratic). MS/MS parameters for phenolic compound determination were as follows: ion source voltage 3 kV, ion source temperature 130 °C, desolvation temperature 350 °C, and desolvation gas flow 600 L/h, with nitrogen as the desolvation gas and argon as the collision gas. MRM transitions for the target analytes were: cyanidin 287.11 → 121.2; gallic acid 168.89 → 124.9, (+)-catechin 291.01 → 138.9, quercetin 301.02 → 150.9. Diode array detection (DAD) was scanning in the 200–400 nm range, with specific wavelengths for each compound (285 nm for cyanidin, 270 nm for gallic acid, 260 nm for (+)-catechin, and 370 nm for quercetin). For ascorbic acid, an isocratic mobile phase of 0.1% FA in methanol (15% *v*/*v*) and 0.1% FA in ultrapure water (85% *v*/*v*) was used at a flow rate of 300 μL/min, using the same chromatographic column as for phenolic compounds. MS/MS parameters for ascorbic acid were ion source voltage 4 kV, ion source temperature 115 °C, desolvation temperature 300 °C, and desolvation gas flow 300 L/h (with nitrogen as the desolvation gas and argon as the collision gas). MRM transition for ascorbic acid was 175.13 → 115.2, and the monitored wavelength was 251 nm.

#### 2.3.2. Total Phenolic Content (TPC)

The TPC was determined by using the Folin–Ciocalteu reagent according to Singleton et al. (1999) [22]. Briefly, 0.2 mL of extract water solution (1 mg/mL) was added to 1 mL of 1:10 diluted Folin–Ciocalteu’s phenol reagent, followed by the addition of 0.8 mL of sodium carbonate solution (7.5% *w*/*v*). After 30 min in the dark at 40 ± 1.0 °C, the absorbance at 750 nm was measured spectrophotometrically (UV-Vis 1601 spectrophotometer, Shimadzu, Milan, Italy). Distilled water was used as a blank. TPC was estimated from a standard curve of gallic acid (R^2^ = 0.999). All measurements were performed in triplicate, and the results were expressed as gallic acid equivalent in µg/mL rosehip extract (µgGAE/mL extract).

#### 2.3.3. Total Flavonoids Content (TFC)

The TFC was assessed using the pharmacopeial method with slight modifications [23]. Briefly, 0.1 mL of 5% (*w*/*v*) AlCl_3_ solution was added to 0.9 mL of the extract solution (1 mg/mL *w*/*v*). After incubation at room temperature for 30 min in the dark, the absorbance was measured at 430 nm. The TFC was calculated based on a standard curve of quercetin (R^2^ = 0.999), and was expressed as µg of quercetin equivalent (µg QE)/mL of extract. All measurements were performed in triplicate.

#### 2.3.4. Extract Antioxidant Activity (AA) Analyzed by DPPH

The AA was assessed using the 2,20-di-phenyl-1-picrylhydrazyl radical (DPPH) reduction assay as reported by Brand-Williams et al. (1995) [24] with slight modifications. In brief, a solution (1 mg/mL) of extract as well as ascorbic acid (used as a standard antioxidant compound) were mixed 1:1 with a solution of DPPH (0.1 mM in methanol) at room temperature. The mixtures were kept in the dark for 30 min, and the absorbance was measured at 517 nm. Methanol was used as a blank solution, and DPPH solution was the control. The test was carried out in triplicate. The results were reported as percentage of inhibition of the DPPH radical according to the following equation: Inhibition % = [(A_0_ − A)/A_0_] where A_0_ was the absorbance of the DPPH control, and A was the absorbance of the sample with DPPH.

### 2.4. Encapsulation of Rosa canina Extract into Lipid Vesicles

#### 2.4.1. Preparation of Glycethosomes (GlyEts)

GlyEts were prepared through the ethanol injection method reported by Ma et al. (2018) [25] with minor modifications. Briefly, Lipoid H50 (200 mg) was dissolved in 4 mL of ethanol under agitation. The *RC* hydroalcoholic extract (40 mg) was dissolved in double-distilled water (13.65 mL) with vegetable glycerol at 85% (2.35 mL). The ethanolic solution of phospholipids was slowly added (1 mL/min) with a syringe to the aqueous solution of *RC* extract under constant stirring at 400 rpm at 30 °C. The resulting vesicle suspensions, namely GlyEts-RC, were homogenized through ultrasonication at 50 Hz for 15 min (Elma Transonic T310, Singen, Germany) to convert large multilamellar vesicles into smaller vesicles. Unloaded glycethosomes (GlyEts) were prepared as a control.

#### 2.4.2. Preparation of Allanthosomes (AllEts)

AllEts were prepared by the ethanol injection method as previously described for GlyEts. Briefly, Lipoid H50 (200 mg) was dissolved in 4 mL of ethanol under agitation. The hydroalcoholic *RC* extract (40 mg) was dissolved in 16 mL of double-distilled water to which allantoin (40 mg) was subsequently added, stirring at 400 rpm at 30 °C. The ethanolic solution of phospholipids was slowly added (1 mL/min) with a syringe to the aqueous solution of extract and allantoin under constant stirring at 400 rpm at 30 °C. The obtained suspension was named AllEts -RC, containing 2 mg/mL of allantoin and *Rosa canina* extract. Unloaded AllEts were prepared as a control.

### 2.5. Vesicles Characterization

#### 2.5.1. Vesicle Size, Polydispersity Index, ζ Potential and pH Measurement

The prepared nanocarriers were characterized in terms of vesicle size (VS) and polydispersity index (PDI). The GlyEts and AllEts suspensions were diluted (1:800 *v*/*v*) in MilliQ water. Average particle size (hydrodynamic diameter) and PDI were measured through a Dynamic Light Scattering (90 Plus Particle Size Analyzer, BTC) while the ζ potential measurements were carried out at 25 °C through a Nicomp™ 380 ZLS instrument after a dilution (1:1000 *v*/*v*). The pH of GlyEts and AllEts suspensions was determined by using a digital pH-meter (Crison Instruments, S.A. Barcelona, Spain).

#### 2.5.2. Encapsulation Efficiency (EE)

To assess the content of bioactive compounds encapsulated within the vesicles, the EE was determined through the dialysis method. Specifically, 1 mL was purified from non-incorporated components through dialysis using Spectra/Por^®^ membranes with a 14 kDa molecular weight cut-off. The dialysis was performed in 0.5 L of water for 2 h at room temperature, with distilled water refreshed after 30 min (2 L in total). At the conclusion of the purification process, the quantification of loaded RC was carried out on 0.2 mL aliquots extracted from within the membrane. These aliquots were then diluted 1:1 *v/v* in methanol, and 0.1 mL was analyzed by adding 0.5 mL of Folin–Ciocalteu reagent (diluted 1:10 *v/v* in water) and 0.4 mL of calcium carbonate (7.5% in water) [26]. The samples were incubated at 40 °C for 20 min and then centrifuged at 14,000 rpm for 10 min. Finally, they were subjected to spectrophotometric analysis, recording the absorbance at λ = 750 nm in the UV-Vis 1601 spectrophotometer (Shimadzu, Milan, Italy). The EE was calculated as a percentage of the rosehip extract after dialysis versus that before dialysis, as in the following formula:

EE% = (RC% dialyzed sample/RC% non-dialyzed sample) × 100

#### 2.5.3. Physical Stability

The physical stability of the GlyEts and AllEts with 20% ethanol was assessed by monitoring the size and the PDI every 7 days for 4 weeks, and then every 30 days for the next 6 months of storage at 4.0 ± 1.0 °C in the dark. For this study, at predetermined time points (0, 7, 14, 21, 28, 60, 90, 120, 150, and 180 days). Aliquots of vesicle suspensions were diluted in ultrapure water (1:800 *v/v*), and the change in nanovesicles size and PDI were measured through DLS as reported in Section 2.5.1.

#### 2.5.4. Accelerated Stability Studies

The accelerated stability of the loaded and unloaded GlyEts and AllEts was performed with the multi-wavelength analytical photocentrifuge LUMiSizer^®^ (L.U.M. GmbH, Berlin, Germany) using STEP-Technology^®^ (Nagpur, India). Samples of lipid vesicles (1 mL) were placed in the cuvette (PA110-135XX, L.U.M. GmbH, Berlin, Germany) and submitted to increasing rotor speeds up to 4000 rpm at 25 °C. The evolution of the transmission profiles, trace instability phenomena, and instability indices were analyzed using SEPView^®^ software 6.4.678.6069.

### 2.6. Cell Toxicity Studies

The human fibroblast WS1 cells were grown in high glucose DMEM, supplemented with 10% fetal bovine serum, 2 mM L-Glutamine, 100 units/mL penicillin, and 100 µg/mL streptomycin, at 37 °C in a humidified atmosphere of 5% CO_2_/95% air. To evaluate the biological effect of the nanovesicles, an MTT assay was performed. Briefly, WS1 cells were seeded in a 96-well plate and incubated overnight to allow cell adhesion. After 24 h, different concentrations of nanovesicles (0.1, 0.05, and 0.025 µg/mL of extract) were added to 10 µL of 5 mg/mL MTT solution (3-(4,5-dimethylthiazol-2-yl)-2,5-diphenyltetrazolium bromide) and incubated for 4 h. Then, the media were replaced with 100 µL of isopropanol to solubilize the formazan crystal. Finally, absorbance at 570 nm was measured with a multimode plate reader (EnSpire Multimode Plate Reader, Perkin-Elmer, Waltham, MA, USA). The sample absorbance at 690 nm was used as reference wavelength for correction. Results are shown as percentage of cell viability compared to untreated control cells (100% viability).

### 2.7. Hemocompatibility Studies

Human blood was collected in accordance with ethical standards, the Declaration of Helsinki, and both national and international guidelines from the Immunohematology and Transfusion Medicine Service of the Bologna Metropolitan Area (Protocol number 0000816 of 23 February 2024). Erythrocytes from human blood were isolated by centrifugation at 116× *g* for 15 min, and subsequently diluted to 5% (*v*/*v*) with PBS. In brief, a biological sample (2 mL) was prepared by adding 1 mL of RC extract (67 μg/mL), loaded and unloaded AllEts or GlyEts diluted in PBS (3.33% *v/v*) to 1 mL (5%) red blood cell (RBC) suspension in isotonic buffer solution (pH 7.4). Triton X100 (0.1% *v/v*) and phosphate-buffer solution were employed as positive and negative controls, respectively. Samples were incubated for 60 min at 37 °C, followed by centrifugation at 1100× *g* for 3 min. The absorbance of the supernatant was measured at 542 nm. The hemolysis rate was calculated using the following equation:

Hemolysis% = [(Abs sample − Abs negative control)/(Abs positive control − Abs negative control)] × 100.

### 2.8. Intracellular Antioxidant Activity

WS1 cells were seeded at a density of 21,000 cells per well. The following day, cells were incubated for 30 min with 50 μM terbutyl hydroperoxide, an inducer of oxidative stress. Subsequently, cells were treated with *RC* hydroalcoholic solution (0.2 mg/mL), nanovesicles, and ascorbic acid solution (0.25 mg/mL) for 6 and 24 h. After washing in phosphate buffer, the fluorescent probe H_2_DCFDA was added at the concentration of 2 μM, in phenol red-free DMEM medium for another 30 min. After washing again, the samples were analyzed by fluorescence microscopy using a Nikon Eclipse TE300 confocal microscope, using λ_exc_ 488 nm and λ_em_ 515 nm. Finally, the fluorescence intensity of the acquired images was measured using FIJI software (v 2.3.0/1.53f) [27].

### 2.9. Wound Dressing Preparation and Vesicles Visualization

The final wound dressing was prepared using Spanish broom as supporting material. Specifically, 100 μL of vesicles prepared, as reported in Section 2.4.1 and Section 2.4.2, were absorbed onto a 1 × 1 cm^2^ peace of Spanish broom for an hour. To visualize the final wound dressing, vesicles were stained with the DiO green fluorescent dye. A total of 1 mL of vesicles was stained with 5 µL of DiO and incubated at 37 °C for 20 min. Subsequently, to remove any excess of dye, nanovesicles were dialyzed with a Visking Tubo Dialysis membrane (Medicell International Ltd., London, UK) with a cut-off size of 14,000 Dalton. The tube was immersed in 500 mL of milli-Q water (external phase), maintained under stirring at 200 rpm, and the water was replaced every 30 min for 4 times. Finally, stained nanovesicles were adsorbed by Spanish broom dressing as previously described, and images were acquired through confocal microscopy (Nikon, Eclispse C1, Nikon instruments S.p.A, Florence, Italy).

### 2.10. Scratch Assay

To evaluate the healing ability of the final wound dressing, 30,000 WS1 cells per well were seeded in a specific 96-well plate (Incucyte Imagelock Plate BA-04855, Sartorius, Göttingen, Germany) and incubated at 37 °C and 5% CO_2_. Once the confluence was reached, the wound area was created using Incucyte^®^ 96-Well Woundmaker following the protocol of the instruction manual. After creating the wound area, detached cells, and cell debris were removed by washing twice with PBS. Finally, in order to monitor the effects on wound closure, 100 µL/well of different conditioned media were added to the cells. Briefly, the different media were obtained by loading a square of Spanish broom dressing (area 2 cm^2^) with 75 μL of pure extract, or vesicle encapsulated extract, or empty vesicles. A complete absorption of the formulations was reached after 15 min. Dressing samples were then placed in 6 mL of RPMI and incubated at 32 °C for 6 h under magnetic agitation. Afterward, the dressing was removed from the medium, and the resulting suspension was centrifuged at 2000 rpm for 10 min. The supernatant was then filtered through a 0.45 μm syringe filter and supplemented to obtain the final extract in a complete medium supplemented with 10% FBS, 2 mM l-Glutamine, 100 units/mL penicillin, and 100 μL/mL streptomycin.

The healing ability was monitored by using the Incucyte live-cell analysis system (Essen BioScience Ltd., Hertfordshire, UK), and images were acquired every 3 h for 2 days. The relative wound closure was calculated through IncuCyte^®^ ZOOM software (v 2022Brev2).

### 2.11. Statistical Analysis

Data were shown as mean ± standard deviation calculated from three independent experiments. Results were analyzed using one-way and two-way ANOVA test using GraphPad Prism software (V6, GraphPad Software, San Diego, CA, USA). Significance was graphically reported as follows: * *p* < 0.05, ** *p* < 0.01, and *** *p* < 0.001.

## 3. Results and Discussion

### 3.1. HPLC-DAD-MS/MS Analysis of Bioactive Compounds in Rosa canina Extracts

The health benefits of *Rosa canina* L., primarily antioxidative and anti-inflammatory effects, are well known, and many papers are already reported in scientific literature [28,29,30]. Therefore, rosehips were selected as a vegetal matrix to treat wound healing, considering their relevant content of bioactive compounds such as flavonoids, tannins, carotenoids, phenolic acid, and fatty and organic acids [31]. Hence, we prepared a hydroalcoholic extract, and its chemical composition was analyzed by HPLC-DAD-MS/MS. The content of the main bioactive compounds extracted from rosehips is reported in Table 1.

Among the analyzed polyphenols, the most abundant compounds were cyanidin and catechin (8.58 ± 0.44 and 8.02 ± 0.58 µg/mL, respectively). Quercetin was detected at a concentration of 3.56 ± 0.32, and a low concentration of gallic acid (0.83 ± 0.15 µg/mL) was found.

The hydroalcoholic extract showed a high amount of ascorbic acid (4.29 ±0.28 µg/mL). These results are similar to our previous work [20], although the content of cyanidin is higher in this extract (8.58 ± 0.44 µg/mL). In addition, comparing our results with data reported by Taneva et al. (2016) [32], a significant difference in ascorbic acid content was observed. In particular, the Bulgarian wild-growing rose hip fruits and Tunisian, Turkish and some commercial Bulgarian rose hip fruits had higher values of ascorbic acid. Probably, this can be attributed to various factors such as agro-ecological conditions, including locality, altitude, humidity, soil composition, rainfall, and temperature, that influence the growth of *Rosa canina* L.

### 3.2. Total Phenolic Content, Total Flavonoid Content, and Antioxidant Activity

The extract obtained from fresh rosehips was characterized by a high content of TPC (115.31 ± 0.59 µg GAE/mL). Thus, the extract could be a potential bioactive agent for providing several beneficial effects in wound healing. Moreover, our results are in agreement with data reported by Paunovic et al. (2019) [33], which showed that the TPC value of *R. canina* fruit extract is 90.51 mg GAE/g sample.

The TFC was determined by an assay based on the ability of the aluminum ion to form a complex with carbonyl and hydroxyl groups of flavonoids. TFC (3.96 ± 0.92 µg QE/mL) was lower than TPC (115.31 ± 0.59 µg GAE/mL). Probably, TPC and TFC variability could be due to the climatic conditions, geographical location, and fertility of the soil where *Rosa canina* L. has grown [34]. Finally, the antioxidant activity (AA) of a 1 mg/mL *RC* ethanolic extract solution was analyzed by DPPH assay. From a pharmaceutical perspective, the analysis of AA of vegetable matrix is essential to define valuable sources of bioactive compounds such as antioxidant [35]. Moreover, Wenzig et al. (2008) [36] reported that the antioxidant activity of extracts is correlated with total, rather than individual, phenolic compounds of rosehips. Hence, *RC* extract showed a high AA (92.45 ± 0.84%) and TPC (115.31 ± 0.59 µg GAE/mL), agreeing with the data reported in the literature.

Based on the results of TPC, TFC, and AA, it can be concluded that bioactive compounds present in *RC* extract could be used for pharmaceutical applications.

### 3.3. Encapsulation of RC Extract into Vesicular Systems

The use of natural extracts is widespread in the food, cosmetic, and pharmaceutical industries due to their efficacy, low toxicity, and natural origin. However, their use is often limited by their instability, as they easily undergo oxidation process, and low solubility. The encapsulation of *RC* extract into vesicular systems is a successful approach to overcome these limitations. Hence, in this study the *RC* hydroalcoholic extract (2 mg/mL) was encapsulated in GlyEts and AllEts, containing 20% EtOH. GlyEts are based on phospholipids hydrated with a mixture of water, glycerol, and ethanol. Glycerol was added due to its moisturizing and cosolvent properties, and ethanol as a penetration enhancer, both helping the active substance pass through the skin [37]. AllEts, in contrast, were prepared using allantoin, which is traditionally used in the treatment of skin ulcers thanks to its capability to remove necrotic tissue, promoting cell proliferation and skin epithelization [38].

Both vesicular systems were prepared by the ethanol injection method, which guarantees many advantages, e.g., simplicity, fast implementation, and reproducibility, as well as the fact that it does not cause lipid degradation or oxidative alterations [39].

### 3.4. Vesicles Characterization

The evaluation of physico-chemical properties of vesicles is crucial to ensure their in vitro and in vivo performance. In particular, for wound treatment, the desirable properties of vesicles are small particle size, particularly below 300 nm, homogeneous (monodisperse) systems, good dispersion stability, and high encapsulation efficiency [37]. Vesicles with small particle size play a significant role in the skin penetration and delivery of the bioactive compounds into the deeper layers of the skin [37]. For this reason, the prepared vesicles were characterized in terms of vesicle size, PDI, pH, *ζ* potential, and entrapment efficiency (EE), and the data are shown in Table 2 and Table 3. The size of unloaded GlyEts and AllEts was below 200 nm, and the PDI value was less than 0.2, suggesting a low tendency to form aggregates and good homogeneity of these vesicular systems. The encapsulation of *RC* extract increased the size of GlyEts and AllEts (207.6 ±13 and 237.3 ± 14.9, respectively). This enlargement could be attributed to the incorporation of bioactive compounds in vesicle structures, as reported in our previous work [21].

Interestingly, *ζ* potential increased significantly from −14.73 ± 4.46 to −29.24 ± 4.66 mV between unloaded and loaded GlyEts (*p* < 0.001). An even greater increase is observed when allantoin was added to the formulation, reaching a *ζ* potential of about −35 mV.

Moreover, it was observed that ζ potential values of loaded GlyEts and AllEts were highly negative (−29.24 ± 4.66 and −37.21 ± 2.62, respectively), indicating the good stability of vesicles. Generally, a ζ potential value of ±30 mV indicates the electrostatic repulsion between vesicles, thus favoring system stability over time.

Concerning the pH, it was observed that pH ranges from 4.18 to 4.9, rendering the formulations suitable for treating wounds. In fact, it is known that healing wounds are characterized by low pH values, around 4.0–6.0, while non-healing wounds have elevated pH values, 7.0 or higher [40].

Finally, the encapsulation efficiency was determined by the dialysis methods and all the tested formulations demonstrated a high EE over 70%. It is worth noting that GlyEts demonstrated an EE of up to 91.0% ± 3.5. In both formulations, EE was probably influenced in a positive way by the concentration of ethanol (usually up to 40%) and phospholipids favoring the distribution of bioactive compounds of *RC* extract between the core and the membrane phospholipid bilayer.

### 3.5. Nanovesicles Stability

The stability of formulations over time is an important condition that becomes essential during the development of an industrial product. Stability tests were carried out with two different instruments: the DLS, which involves measuring size over time, and the LUMiSizer^®^, which provides a reliable stability projection from the measurement of sedimentation/creaming.

**Figure 1:**
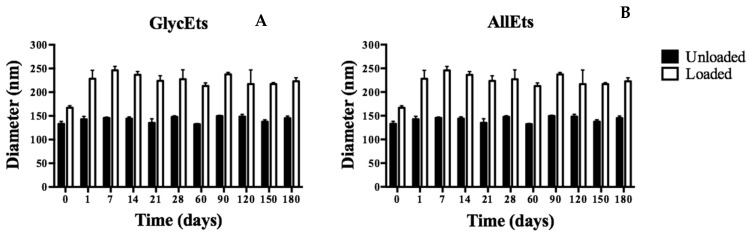
Study of stability over time. The formulations were stored at 4 °C for six months and analyzed by DLS at different times. Stability measurements of GlyEts (**A**) and AllEts (**B**).

For the stability analysis with DLS, the formulations were stored at 4.0 ± 1.0 °C and analyzed at regular time points up to 6 months. In Figure 1, it is possible to observe a slight increase in the size of both formulations in the first week, likely due to fusion or aggregation phenomena, after which they remain almost stable over time.

**Figure 2:**
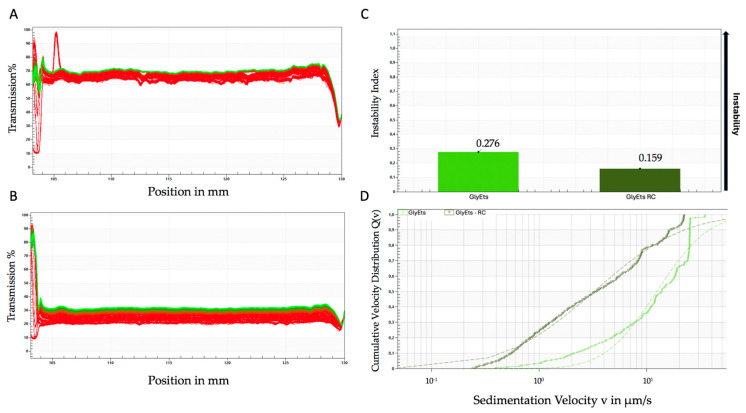
Accelerated stability analyzed by the Lumisizer photocentrifuge. Transmittance of unloaded (**A**), and loaded GlyEts (**B**), the first transmission profile is shown in red, while the last is shown in green. Instability index over time (**C**), and sedimentation rate distribution (**D**). The graphs are representative of one of three different preparations with the same results.

Accelerated stability tests were also performed using the LUMiSizer^®^ photocentrifuge [41]. Figure 2A,B shows the extinction/transfer profiles of unloaded and loaded GlyEts. The graphs shown represent the transmittance (*y*-axis) along the height of the test tube (*x*-axis) over time (red represents the start of the test, green represents the end of the test). From these, it can be observed that unloaded GlyEts separate faster than loaded GlyEts, indicating less stability over time. Interestingly, it is clear that the formulations have a different base transmittance, probably due to the encapsulation of the extract making the formulation less transparent. Moreover, from the instability values shown in Figure 2C, it appears that the unloaded GlyEts deposit almost immediately compared with the loaded ones. The instability value obtained analyzing the samples after 15,000 s results in about 0.276 and 0.159, respectively, for the unloaded and loaded formulations.

**Figure 3:**
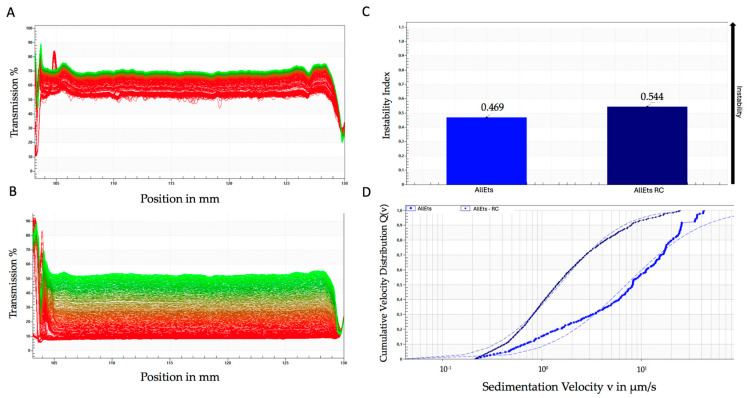
Accelerated stability using the LUMiSizer^®^ photocentrifuge. Transmittance of unloaded (**A**), and loaded AllEts (**B**), the first transmission profile is shown in red, while the last is shown in green. Instability index over time (**C**), and sedimentation rate distribution (**D**). The graphs are representative of one of the batches prepared that demonstrated overlapping results.

It is worth noting that AllEts show a similar sedimentation profile, although the variability is more pronounced, especially for the loaded formulation (Figure 3A,B). As shown for GlyEts, the loaded preparations exhibit a lower sedimentation rate with respect to the unloaded ones, again confirming the good stability of the formulations (Figure 3C,D). Notably, the instability index was higher than that of GlyEts. However, both formulations exhibited good values corresponding to 0.469 for unloaded and 0.544 for loaded AllEts (Figure 3C), according to Zielińska et al. (2018) [41].

### 3.6. Cell Toxicity

The biocompatibility, which is the non-toxicity on cells and tissues, is one of the main requirements of pharmaceutical formulations for wound healing. For this reason, the study of biological activity began with an MTT metabolic assay on the human fibroblast cell line WS1. It is known that the wound healing process takes place through the activity of various cell types, such as platelets, neutrophils, monocytes, macrophages, fibroblasts, keratinocytes, endothelial cells, epithelial cells, and myofibroblasts. However, among the aforementioned cells, fibroblasts have long been recognized as key cells in wound healing process, as they play a major role in all the phases of wound healing [42].

**Figure 4:**
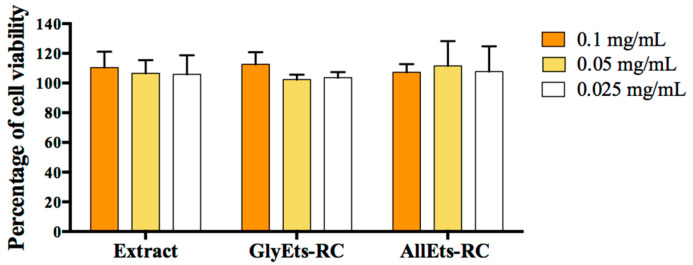
Cytotoxicity measurements. WS1 cells were seeded and treated with different concentration of *RC* extract, GlyEts and AllEts RC (0.1, 0.05, 0.025 mg/mL of extract). After 24 h treatment, cell viability was analyzed by the MTT assay.

WS1 cells were treated with *RC* extract, loaded and unloaded GlyEts or AllEts. The data shown in Figure 4 revealed the absence of toxicity for all extract concentrations tested (0.1, 0.05, and 0.025 mg/mL). Additionally, none of the three concentrations of loaded and unloaded formulations showed signs of cytotoxicity even at the highest concentrations. This allows the use concentration to be adjusted according to different possible applications.

### 3.7. Hemocompatibility Studies

Hemocompatibility describes the interaction between a foreign substance and blood, with minimal adverse effects. Hemocompatibility is also a critical requirement for the safe application of medical devices in wound healing [43]. National safety standards state that the hemolysis rate must be less than 5% for a formulation to be deemed biosafe.

**Figure 5:**
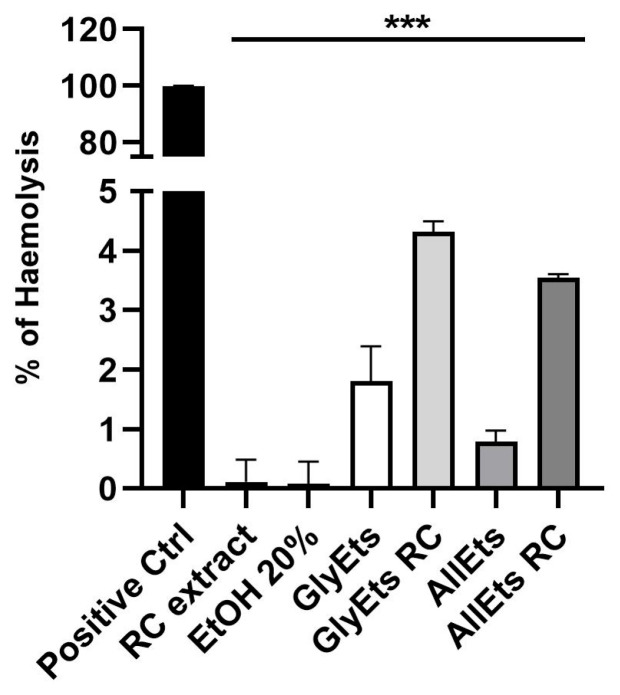
Biosafety Assay. Hemolytic rate (%) after treatment with *RC* extract, a solution of ethanol 20%, unloaded and loaded GlycEts or AllEts (*** *p* < 0.0001 between all samples under the black line compared to the positive control obtained treating sample with Triton X100).

Therefore, we tested the hemocompatibility of the nanoformulations (Figure 5). The results indicate that *RC* extract, as well as unloaded and loaded GlyEts and AllEts, are biosafe, exhibiting a hemolysis rate of less than 4.5%, confirming their blood compatible nature.

### 3.8. Intracellular ROS Determination

The different stages of the wound healing process require a delicate balance between pro-oxidant and antioxidant agents. The normal physiology of wound healing involves low levels of ROS and oxidative stress, while overexposure to oxidative stress correlates with slowed or impaired healing. Antioxidants, in general, are believed to help control oxidative stress in the wound and thus accelerate wound healing.

**Figure 6:**
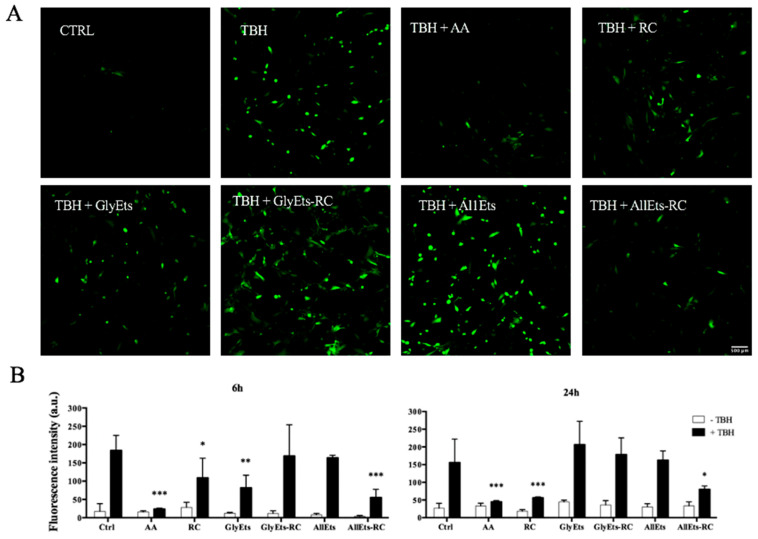
Antioxidant Activity. Oxidative stress was induced in WS1 cells with TBH and the antioxidant activity was measured after addition of different formulations for 6 and 24 h. (**A**) Representative fluorescence confocal images of WS1 cells treated with the nanocarriers. (**B**) Fluorescence intensity quantification. Significance was graphically reported as follows: * *p* < 0.05, ** *p* < 0.01, and *** *p* < 0.001.

Therefore, oxidative stress was initially induced in WS1 cells with TBH, then the different formulations were added for 6 and 24 h. Finally, intracellular ROS levels were determined using H_2_DCFDA. Representative fluorescence images are shown in Figure 6A, while Figure 6B illustrates the quantification of fluorescence intensity. Ascorbic acid, which serves as a positive control, and hydroalcoholic extract of *RC* exhibited a significant reduction in oxidative stress after only 6 h of treatment. Interestingly, among the formulations studied, AllEts-RC showed the most consistent effect, reducing oxidative stress by approximately 50%. Probably, the observed efficacy was due to the positive effect of allantoin, which has own antioxidant properties [44], on the total antioxidant activity of AllEts-RC, which is clearly evident at 24 h.

### 3.9. Impregnation of Spanish Broom Dressing

**Figure 7:**
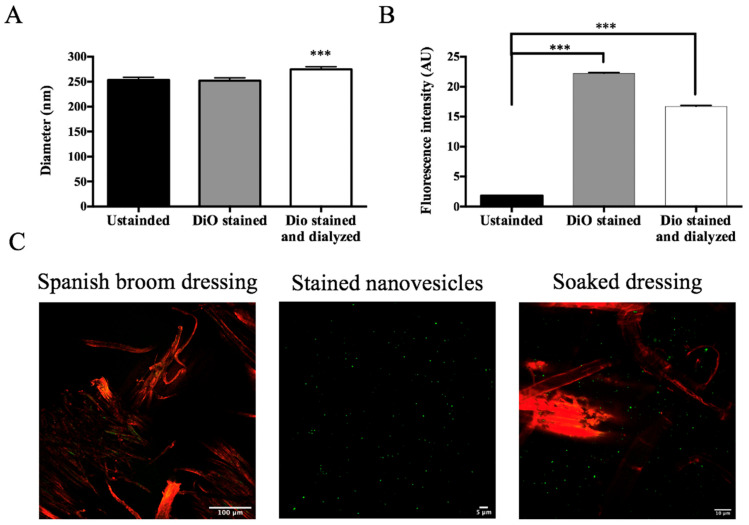
Confocal Analysis of Spanish broom dressing impregnation. (**A**) Size of GlyEts unstained, and stained with DiO, pre and post dialysis: (**B**) fluorescence intensity of GlyEts unstained, and stained, pre o post dialysis; (**C**) representative confocal images. Spanish broom fibers (red) and GlyEts (green). Significance was graphically reported as *** *p* < 0.001.

Currently, several biopolymer products suitable for skin dressings are commercially available. In this work, we tested a Spanish broom gauze impregnated with nanovesicular formulations as an alternative to cotton gauzes. To visualize the dressing impregnation, GlyEts were stained with the fluorescent probe DiO and images were acquired under confocal microscopy. Initially, the nanovesicles were stained and then dialyzed to remove any unbound dye. Nanocarrier sizes were determined, and a minimal variation in dimensions was observed (Figure 7A). The effective labeling of the nanocarriers was evaluated by measuring the fluorescence intensity by means of the FluoroSkan Ascent FL Labsystem (Figure 7B). A substantial increase is evident after staining, followed by a slight decrease post-dialysis. Finally, Spanish broom dressings were loaded with stained nanocarriers to study the final product (Figure 7C). The structure of Spanish broom and nanovesicle distribution can be observed. Interestingly, the nanocarriers appear homogeneously dispersed among the fibers, suggesting successful deposition on the gauze. Hence, this innovative wound dressing would give the consumer a green, biocompatible, and safe option once it was put on the skin. Similar results were observed for AllEts.

### 3.10. Scratch Assay

**Figure 8:**
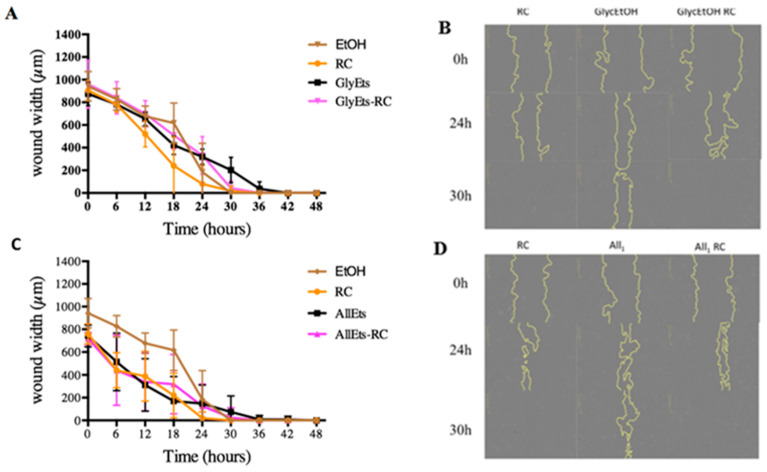
Wound healing effect. Monitoring of wound width during the 48 h treatment. (**A**) Effect on wound closure of RC extract, GlyEts, and GlyEts-RC. (**B**) Representative phase images over time. (**C**) Effect of RC extract, AllEts and AllEts-RC. (**D**) Representative phase images over time.

It is widely reported that two essential processes must take place during wound healing: cell proliferation and migration. Having verified the absence of toxicity of the formulations and the regular proliferation of fibroblasts, it was therefore considered important to analyze cell migration. For this purpose, the scratch test, one of the most widely used methods [45], was applied for assessing the collective migration of cells in vitro in two dimensions. Fibroblasts were treated with a culture medium in which Spanish broom gauze impregnated with the loaded GlyEts-RC had been soaked for 6 h. Scratch was made in each well to simulate the wound, and the time required for its complete closure was then evaluated. This monitoring was conducted using the Incucyte platform, which allows the automated acquisition of images at different times. It can be observed that all of the conditions resulted in a scratch closure within 48 h (Figure 8). Specifically, pure *RC* extract leads to wounds closing after about 30 h. Regarding GlyEts, it is interesting to note that only cells treated with loaded formulation reach the complete wound closure at the same time (Figure 8A,B). On the contrary, in the case of cells treated with the unloaded GlyEts, at least 40 h are required.

Similar results were obtained with AllEts. After 30 h, wound closure is reached with the *RC* extract as well as with the loaded formulations (Figure 8C,D). Our results are in line with a body of work evaluating the wound healing properties of rose hips, showing their potential benefits in accelerating the healing process and promoting tissue regeneration [46].

## 4. Conclusions

Currently, the use of plant extracts remains a promising strategy in the treatment of wounds. Therefore, in this work, *Rosa canina* extract, rich in polyphenols and ascorbic acid, has been successfully encapsulated into GlyEts and AllEts, respectively. GlyEts are based on phospholipids hydrated with a mixture of water–glycerol and ethanol, while AllEts were prepared using allantoin. Both nanovesicles with a size below 300 nm, high encapsulation efficiency (>70%), and long-term stability successfully protected bioactive compounds from oxidation. Moreover, vesicles are biosafe and non-cytotoxic on the human fibroblast WS1. Finally, Spanish broom gauzes impregnated with the GlyEts or AllEts suspension containing *RC* extract led to complete closure of the scratch in about 30 h. Our results represent a promising proof of concept to develop alternative wound dressings based on natural bioactive compounds and Spanish broom fibers able to reduce wound inflammation and oxidative stress. Further studies are required to validate the ability of these wound dressings to improve the healing process in vivo.

## Figures and Tables

**Table 1:** Content of ascorbic acid and polyphenols from *Rosa canina L*. extract.

Anthocyanidins *	Flavonoids *		Benzoic Acid *	Vitamins *
Cyanidin	Catechin	Quercetin	Gallic Acid	Ascorbic acid
8.58 ± 0.44	8.02 ± 0.58	3.56 ± 0.32	0.83 ± 0.15	4.29 ±0.28

* Data are reported as µg/mL in a solution 1 mg/mL of extract.

**Table 2:** Size, PDI, pH, *ζ* potential and EE% of GlyEts, unloaded and loaded with *RC* extract.

Sample	Size (nm)	PDI	pH	ζ (mV)	EE%
**GlyEts**	144.0 ± 9.7	0.174 ± 0.028	4.27 ± 0.01	−14.73 ± 4.46	/
**GlyEts-RC**	207.6 ± 13	0.173 ± 0.041	4.18 ± 0.07	−29.24 ± 4.66	91.0 ± 3.5

**Table 3:** Size, PDI, pH, *ζ* potential and EE% of AllEts, unloaded and loaded with *RC* extract.

Sample	Size (nm)	PDI	pH	ζ (mV)	EE%
**AllEts**	146.6 ± 3.2	0.167 ± 0.018	4.9 ± 0.12	−33.40 ± 2.74	/
**AllEts** ***-RC***	237.3 ± 14.9	0.191 ± 0.041	4.24 ± 0.08	−37.21 ± 2.62	72.9 ± 4.8

## Data Availability

The original contributions presented in this study are included in the article. Further inquiries can be directed to the corresponding author.
